# Depletion of c-Rel from Cytokine Gene Promoters Is Required for Chromatin Reassembly and Termination of Gene Responses to T Cell Activation

**DOI:** 10.1371/journal.pone.0041734

**Published:** 2012-07-30

**Authors:** Fiona S. Poke, William R. Upcher, Owen R. Sprod, Arabella Young, Kate H. Brettingham-Moore, Adele F. Holloway

**Affiliations:** 1 Menzies Research Institute Tasmania, University of Tasmania, Hobart, Tasmania, Australia; 2 School of Medicine, University of Tasmania, Hobart, Tasmania, Australia; Aligarh Muslim University, India

## Abstract

The role of the Nuclear Factor κB (NF-κB) transcription factor family in T cell function has been well described. The c-Rel family member is of particular importance in initiating T cell responses to antigen and regulating activation of inflammatory cytokine genes, including the Interleukin-2 (IL-2) and Granulocyte macrophage colony stimulating factor (GM-CSF) genes. c-Rel is required for chromatin remodeling of these gene promoters, which involves depletion of histones from the promoters in response to T cell activating signals. These chromatin remodeling events precede transcriptional activation of the genes. The subsequent down-regulation of cytokine gene expression is important in the termination of an immune response and here we examine this process at the murine GM-CSF and IL-2 genes. We show that the cytokine mRNA levels rapidly return to basal levels following stimulus removal and this is associated with reassembly of histones onto the promoter. Histone reassembly at the GM-CSF and IL-2 promoters occurs concomitantly with depletion of RelA, c-Rel and RNA polymerase II from the promoters. Furthermore we show that transcriptional down-regulation and chromatin reassembly is dependent on depletion of c-Rel from the nucleus, and that this is regulated by the nuclear translocation of the NF-κB inhibitor, IκBα. The nuclear activation of c-Rel therefore not only regulates the initiation of GM-CSF and IL-2 gene activation in response to T cell activation, but also the termination of these gene responses following the removal of the activating signal.

## Introduction

The rapid activation and silencing of immune genes is essential for normal immune function. While much emphasis has been placed on how these genes are switched on, equally important is how these gene responses are terminated, as the appropriate resolution of an immune response is essential to prevent unnecessary inflammation and autoimmune responses. A key player in regulating the activation of inducible genes that are important mediators of the immune response is the Nuclear Factor κB (NF-κB) transcription factor family. However less is known about how these transcription factors are regulated during the resolution of an immune response. The NF-κB family consists of five members, p50 (NF-κB1), p52 (NF-κB2), RelA (p65), RelB and c-Rel, which play an important role in regulating inducible gene expression in the immune system [1]. The NF-κB proteins contain a common region called the Rel Homology Domain that binds to DNA, and all except p50 and p52 also contain distinct transactivation domains [1]. The classical model of NF-κB activation involves NF-κB proteins bound to inhibitory IκB proteins in the cytoplasm [2]. In response to signals that activate the IκB kinase, IKK, IκB is phosphorylated then degraded via the proteasome, allowing rapid translocation of NF-κB to the nucleus. The NF-κB proteins then bind as homo- or heterodimers to DNA elements in gene promoters regulating gene transcription.

The NF-κB family members have distinct physiological roles, as evidenced by knockout mouse studies [3]. While knockout of RelA is embryonic lethal, knockout mice for other family members are viable, but have immunological defects. The immune system appears to develop normally in c-Rel^−/−^ animals, but the mice display immunodeficiencies due to abnormalities in T, B, macrophage and dendritic cell function [3]. Splenic T cells from c-Rel^−/−^ mice have reduced proliferative ability [4], resulting from reduced IL-2 gene expression following activation of the T cell receptor and costimulatory molecule. In addition, expression of the inflammatory cytokines GM-CSF and IL-3 are also reduced in these cells [4], [5]. Two NF-κB binding sites are found within the IL-2 promoter, and bind both RelA and c-Rel. Similarly the GM-CSF promoter contains two NF-κB binding sites, a classical site that binds RelA and p50 and a variant site that binds RelA and c-Rel [6].

Transcription factors must operate within the chromatin landscape, which can regulate their ability to access their binding sites and drive transcription [7]. While NF-κB proteins are rapidly translocated into the nucleus in response to activating signals, NF-κB dependent genes can be activated with vastly different kinetics. Saccani *et al*. analyzed transcriptional activation of NF-κB dependent genes in macrophages and classified these genes into two broad groups: those that bind NF-κB with rapid kinetics and are activated quickly and those that bind NF-κB more slowly and are activated with delayed kinetics [8]. More recent studies suggest that these may represent genes that do not require chromatin remodeling for activation, compared to genes that do require chromatin remodeling and are therefore activated with slower kinetics [9]. However, transcription factors may also be drivers of these chromatin remodeling events, by recruiting proteins that reorganize the local chromatin environment. In keeping with this, at least for some of its target genes, c-Rel is required for chromatin remodeling events at the gene promoter that are essential for gene activation.

The NF-κB transcription factors are required for activation of both the GM-CSF and IL-2 cytokine genes in response to T cell activation [10]. However, c-Rel, but not RelA, is required for chromatin remodeling events that accompany activation of both the GM-CSF [11] and IL-2 [5] genes. These chromatin remodeling events involve depletion of histones specifically from the promoter region of the gene [11], [12], [13]. Alterations to the NF-κB binding sites have been shown to prevent chromatin remodeling events in a transgenic mouse model [14] and inhibition of NF-κB translocation in mouse T cell lines also prevents promoter chromatin remodeling [11], [15]. Furthermore, GM-CSF gene expression [16], [17] and promoter chromatin remodeling [11] is inhibited in primary CD4^+^ T cells from c-Rel^−/−^ knockout mice. IL-2 expression is also reduced in these cells and chromatin remodeling events are also inhibited in the absence of c-Rel [4], [18]. Similarly, recent studies investigating the role of c-Rel in regulatory T cell (Treg) development and function suggest that c-Rel drives chromatin changes at some target genes in Tregs [19]. c-Rel drives Treg development and function through its regulation of the Foxp3 transcription factor [20], [21], [22], which is critical for their differentiation and function.

Here we present data that suggests that c-Rel maintains the GM-CSF and IL-2 genes in an accessible state following T cell activation. While the chromatin changes that facilitate activation of inducible genes are now relatively well understood, comparatively little is known about how the chromatin environment at gene promoters contributes to transcriptional repression following removal of the activating signal. Here we examine transcriptional down-regulation of the GM-CSF and IL-2 genes in T cells following removal of the activating stimulus. We show that histones are re-deposited at the GM-CSF and IL-2 promoters following removal of the activating stimulus and this occurs concomitantly with loss of RNA polymerase II. Transcriptional down-regulation of the GM-CSF and IL-2 genes does not depend on nucleosome reassembly, as the decrease in mRNA levels precedes histone re-deposition at the promoter. However, the GM-CSF promoter remains hyper-responsive to restimulation until histone reassembly has occurred. Chromatin reassembly is independent of the cell cycle but is dependent on displacement of c-Rel from the gene promoters. We show that upon stimulus withdrawal IκBα accumulates in the nucleus, and is targeted to the GM-CSF promoter with displacement of c-Rel dependent on nuclear accumulation of IκBα.

## Materials and Methods

### Cell Culture

**Figure 1:**
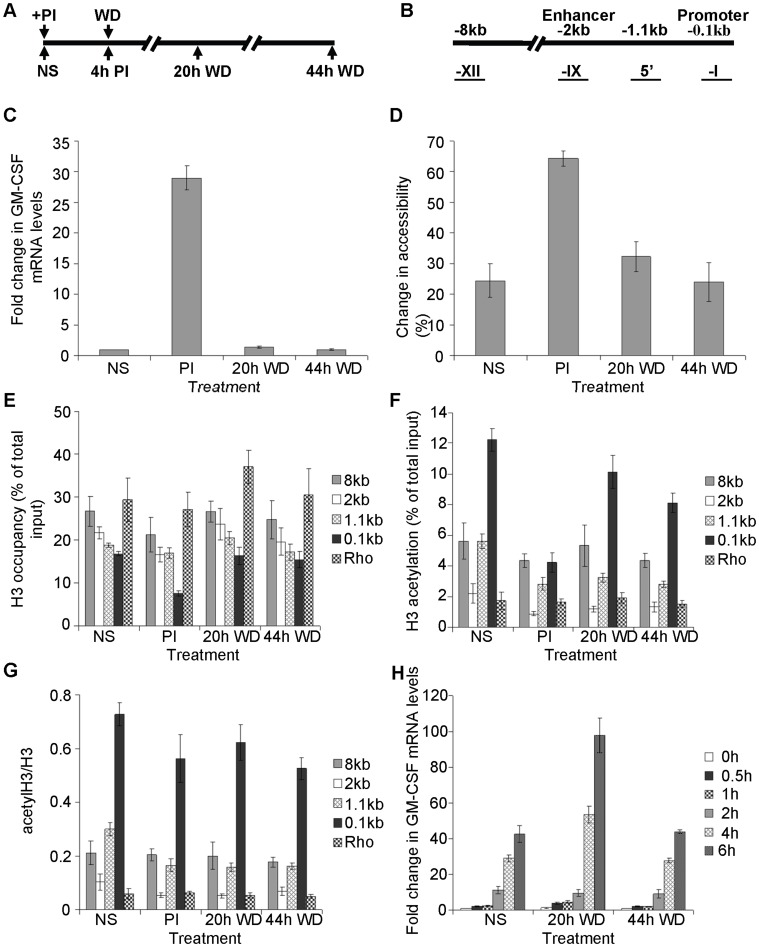
Histones are reassembled at the GM-CSF promoter following stimulus withdrawal. (A) Time course and sampling strategy used for the experiments. Samples were taken from unstimulated (NS) EL-4 T cells, from cells simulated for 4 h with PI (PI), and from cells 20 h and 44 h after withdrawal of the stimulus (20 h WD and 44 h WD). (B) Schematic depicting the position of GM-CSF primer sets used in PCR amplifications. (C–D) GM-CSF mRNA levels relative to GAPDH were determined by RT-qPCR (C) and promoter accessibility to MNase was determined by CHART-PCR (D), in EL-4 T cells treated as indicated. (E–F) Histone H3 (E) and acetylated H3 (F) levels were determined by ChIP analysis at the indicated genomic regions as shown in (B), in EL-4 T cells treated as indicated. (G) The ratio of acetylated H3 to total H3 as determined in (E) and (F) is depicted. (H) EL-4 T cells were either left unstimulated (NS) or treated for 4 h with PI, then the stimulus withdrawn for 20 h (20 h WD) and 44 h (44 h WD). GM-CSF mRNA levels were then determined by RT-qPCR following stimulation with PI for the indicated times. The mean and standard error of three independent experiments is shown in each case (C–H).

Murine EL-4 T cells obtained from American Tissue Culture Collection were cultured as described previously [11], [13]. To induce GM-CSF and IL-2 gene transcription, cells were treated with 20 ng/mL phorbol 12-myristate 13-acetate (PMA; Sigma-Aldrich, USA) and 1 µM calcium ionophore (I; Sigma-Aldrich, USA). The stimulus was removed by washing cells twice in phosphate buffered saline (PBS) and replenishing with fresh medium. The experimental time course and sampling strategy is shown in Figure 1A. When required cells were treated with 10 µg/mL cycloheximide (CHX; Calbiochem, USA), 6 mg/mL pentoxifylline (PTX), 20 nM Leptomycin B (LMB), 10 mM lithium chloride (LiCl), 5 mM BAY 11-7082 and 1 µM MG132 (all from Sigma-Aldrich, USA).

### Gene Expression Analysis

RNA was extracted using Tri-reagent (Sigma-Aldrich, USA), treated with DNase I (Sigma-Aldrich, USA) and cDNA synthesized using Superscript III reverse transcriptase (Invitrogen, USA), as described previously [13]. Quantitative PCR was performed using the QuantiTect SYBR Green PCR kit (Qiagen, USA) in a final volume of 25 µl, including 50 ng cDNA and 0.3 µM of each primer on a Rotor-Gene 6000 real-time cycler (Corbett Research, Australia). Cycling conditions were as follows: 95°C 15 min; 40 cycles of 94°C 15 s, 60°C 60 s. PCR was conducted using primer sets for GM-CSF (+II) and GAPDH, as described previously [13], and IL-2 [23]. Threshold Ct values were converted to copy number from standard curves and data was normalized to GAPDH mRNA levels.

### Chromatin Accessibility by Real-time PCR (CHART-PCR)

DNA accessibility was determined using chromatin accessibility by real-time PCR (CHART-PCR) described by Rao et al [23]. Briefly, cell nuclei (5×10^6^ per 100 µL) were digested with 25 U micrococcal nuclease (MNase; Roche Boehringer Mannheim, Germany) for 5 min at 20°C to determine DNA accessibility. Undigested control nuclei were analyzed alongside to monitor endonuclease activity. DNA was isolated using the QIAamp blood kit (Qiagen, USA) and analyzed using quantitative PCR, as detailed above, with primer set –I, which amplifies a region of the GM-CSF promoter, as described previously [13] and primer set B which amplifies a region of the IL-2 promoter [23].

### Chromatin Immunoprecipitation (ChIP) Analysis

DNA-protein interactions were examined by ChIP analysis, as described previously [24]. Briefly, cells were treated with 1% formaldehyde to crosslink proteins and DNA, and quenched with 0.125 M glycine. Cells were lysed and DNA sheared into 100–500 bp fragments by sonication. The solute was pre-cleared for 1–2 h with salmon sperm DNA/protein A-agarose. Solubilized chromatin was immunoprecipitated using the following antibodies: anti-H3 (1791 Abcam, USA), anti-acetyl H3 (06–599 Millipore, USA), anti-RNA polymerase II (5408 Abcam, USA), anti-c-Rel (sc-71x Santa Cruz Biotechnology, USA), anti-RelA (sc-372 Santa Cruz Biotechnology, USA) and anti-IκBα (Abcam). Immune complexes were recovered using salmon sperm DNA/protein A-agarose for 2 h, washed and eluted. Cross-links were reversed with 0.2 M NaCl and proteins degraded with proteinase K treatment overnight. DNA was purified by phenol/chloroform extraction, ethanol precipitated and resuspended in 50 µL MilliQ water. DNA was amplified using quantitative PCR as detailed above, and levels determined as a percentage of the total input DNA. No antibody control immunoprecipitates were analyzed in parallel. Data for the c-Rel, RelA and RNA polymerase II ChIP analysis was expressed relative to the promoter of a non-expressed gene, rhodopsin. Primer sets designed to amplify a region of the GM-CSF promoter (primer set –I, [13]), a region 1.1 kb upstream of the transcription start site (set 5′; [13]), a region 2 kb upstream of the transcription start site and located within the GM-CSF enhancer (set -IX, sense 5′ AGCAAGGCTGTCTGATGCTA 3′, antisense 5′ AAAGATGACATCAGGGTGGAG 3′), a region 8 kb upstream of the transcription start site (set -XII; sense 5′ CTCATATGGAAGGCCCAAGT 3′, antisense 5′ GGAGCTACAGGCAGTTGTGA 3′), a region of the IL-2 promoter (primer set B, [23]) and a region of the rhodopsin promoter (sense 5′ ATATCTCGCGGATGCTGAAT 3′, antisense 5′ GACAGAGACCAAGGCTGCTT 3′) were used for PCR amplification (Figure 1B).

### Western Blotting

Nuclear extracts were prepared by a modification of the method of Schreiber et al [25], as described previously [11]. Nuclear proteins were resolved by SDS PAGE through 12% polyacrylamide, transferred to nitrocellulose and subjected to western analysis with the following antibodies: anti-c-Rel (sc-71), anti-RelA (sc-372), anti-Sp1 (sc-59, Santa Cruz Biotechnology, USA) and anti-IκBα (Abcam, USA). Proteins were visualized using the Supersignal West Pico Chemiluminescent kit (Pierce, USA).

### Cell Synchronization

EL-4 T cells were synchronized using a double thymidine block. Cells were treated with thymidine (Sigma-Aldrich, USA) at a final concentration of 2.5 mM for 16 h. Cells were washed with PBS and then released into fresh medium for 14 h. Cells were treated a second time with thymidine at 2.5 mM final concentration for 10 h, and then washed with PBS and released into fresh medium.

### Flow Cytometry

Flow cytometry was used to monitor cell cycle based on DNA content of the cells. Harvested cells were washed once in PBS and fixed in 70% ethanol overnight. Prior to flow cytometry, cells were pelleted and resuspended in PBS containing RNase (Qiagen, USA) and stained with 40 µg propidium iodide (Sigma-Aldrich, USA).

### Statistical Analysis

Data were analysed using a paired Student’s t-test with two-tailed distribution.

## Results

### 

#### Histones reassemble at the GM-CSF promoter during transcriptional down-regulation

GM-CSF mRNA is present at very low levels in unstimulated CD4+ T cells or the murine EL-4 T cell line, but levels rapidly increase upon stimulation with the phorbol ester, PMA, and calcium ionophore (PI), which activate the PKC and calcium signaling pathways respectively, and mimic T cell receptor activation. Treatment of EL-4 T cells with PI for 4 h increases GM-CSF mRNA levels by approximately 30 fold, as determined by quantitative RT-PCR (Figure 1C) and withdrawal of this activating stimulus results in a decline in mRNA levels, returning to near basal levels within 20 h of stimulus withdrawal. We have previously shown that increased GM-CSF gene expression in T cells is accompanied by increased accessibility of the GM-CSF promoter, involving depletion of histone H3 from the promoter region, as determined by reduced H3 binding at the promoter detected by ChIP assay [11], [13], [15]. To determine whether the decline in GM-CSF mRNA levels following stimulus removal is accompanied by the return of histones to the promoter, accessibility of the promoter to micrococcal nuclease (MNase) was examined using a PCR-based assay [CHART-PCR; 23]. EL-4 T cells were treated as before, nuclei isolated, incubated with MNase and genomic DNA isolated and analyzed by quantitative PCR with primer set –I, which amplifies a region of the GM-CSF promoter (Figure 1B). The accessibility of the promoter was assessed by comparing the amount of PCR product generated from the digested DNA with the amount generated from undigested DNA. As seen previously [11], [15], in unstimulated EL-4 T cells the GM-CSF promoter displayed some inherent accessibility (approximately 24%) and upon stimulation with PI for 4 h accessibility increased (to approximately 65%, Figure 1D). Following stimulus withdrawal, promoter accessibility declined to approximately 32% within 20 h and returned to basal levels (24%) by 44 h post-stimulation (Figure 1D).

To verify that chromatin disassembly and reassembly is occurring at the GM-CSF promoter in response to cell stimulation and following withdrawal of the stimuli, ChIP analysis was used to determine histone occupancy at the promoter (Figure 1E). The level of promoter H3 occupancy was determined by analyzing DNA immunoprecipitated with anti-H3 antibodies, by quantitative PCR. H3 levels were determined at the promoter using primer set –I and compared to regions 1.1 kb, 2 kb (the enhancer) and 8 kb upstream of the promoter (Figure 1B), as well as to the promoter of the inactive rhodopsin gene. In unstimulated cells, lower levels of H3 were detected at the GM-CSF promoter in comparison to the upstream regions and the rhodopsin promoter, indicative of lower histone density and higher DNA accessibility (Figure 1E). The level of H3 occupancy increased linearly with distance upstream of the promoter (linear regression, P<0.002).

Basal H3 occupancy at the GM-CSF promoter of approximately 17% decreased to 8% upon stimulation with PI for 4 h, demonstrating depletion of histones from the promoter in response to stimulation (P<0.01). Following withdrawal of the activating stimulus, H3 occupancy increased at the promoter returning to basal levels by 20 h (Figure 1E). The GM-CSF enhancer (2 kb; Figure 1E) also showed evidence of chromatin remodeling upon stimulation with PI, as has been shown previously [26], with a significant reduction in H3 occupancy detected following stimulation (P<0.02). However, the histone depletion observed at the enhancer was reduced compared to that seen at the promoter. As with the promoter, histone occupancy returned to basal levels following withdrawal of the stimulus (20 h withdrawal; Figure 1E).

Histone acetylation levels were also examined across the same regions by ChIP analysis with anti-acetyl H3 antibodies. Basal levels of histone H3 acetylation were higher at the GM-CSF promoter compared to upstream regions, as observed previously [13] and particularly compared to levels at the promoter of the rhodopsin gene (Figure 1F). The GM-CSF enhancer (2 kb), displayed low acetylation levels relative to surrounding regions, comparable to that of the rhodopsin promoter (Figure 1F).

Acetylated H3 levels decreased at the GM-CSF promoter after 4 h of PI stimulation, corresponding to the loss of histone H3 (Figure 1E). Following stimulus removal for 20 h and 44 h, elevated levels of acetylated H3 were again detected at the promoter. However, there was a significant difference in acetylation levels at 44 h compared to the unstimulated cells (P<0.003). The variation in levels of acetylated H3 over the time course could potentially be attributed to changes in H3 occupancy. Therefore acetylated H3 levels were normalized to H3 occupancy (Figure 1G). Acetylated H3 levels, relative to total H3 levels, did not vary following stimulation, or 20 h post-stimulus withdrawal, although they were decreased at the GM-CSF promoter 44 h post-stimulation, compared to basal levels (Figure 1G, P<0.003).

To investigate resetting of the promoter chromatin further the response of the GM-CSF promoter to restimulation was examined at 20 h and 44 h post-stimulus withdrawal. Cells re-stimulated at 44 h after withdrawal of the initial stimulus produced the same GM-CSF mRNA levels as cells stimulated for the first time and with identical kinetics (Figure 1H), suggesting the promoter had been reset to the basal state. However, cells that were re-stimulated after only 20 h of stimulus withdrawal displayed increased GM-CSF mRNA levels, suggesting that the promoter was more responsive to re-stimulation (Figure 1H), reflecting that the promoter chromatin still displayed some increased chromatin accessibility at this time.

Put together these data demonstrate that histones depleted from the GM-CSF promoter upon stimulation are reassembled at the promoter following stimulus removal.

#### Histone reassembly at the GM-CSF promoter following stimulus withdrawal is independent of the cell cycle

EL-4 T cells are rapidly dividing cells, completing a cell cycle approximately every 18 h. This time frame is similar to the time needed to begin resetting the GM-CSF promoter chromatin following stimulus withdrawal, as determined above. Therefore the possibility that nucleosome reassembly at the GM-CSF promoter occurs as part of the cell cycle, either through DNA replication or cell division, was explored. A synchronized T cell population was used to investigate nucleosome reassembly and cell cycle stage concomitantly, and was compared to an asynchronized T cell population. The cell cycle stage of both cell populations was determined using flow cytometry of cells stained for DNA content. Synchronization of cells using a double thymidine block resulted in 82% of cells at the G1/S border compared to 50% in the asynchronized population (data not shown). Cells were stimulated for 4 h, the stimulus withdrawn and progression of the cells through the cell cycle monitored. The synchronized cells had completed S phase (DNA replication) within 8 h and cell division within 20 h of withdrawal of the stimulus.

**Figure 2:**
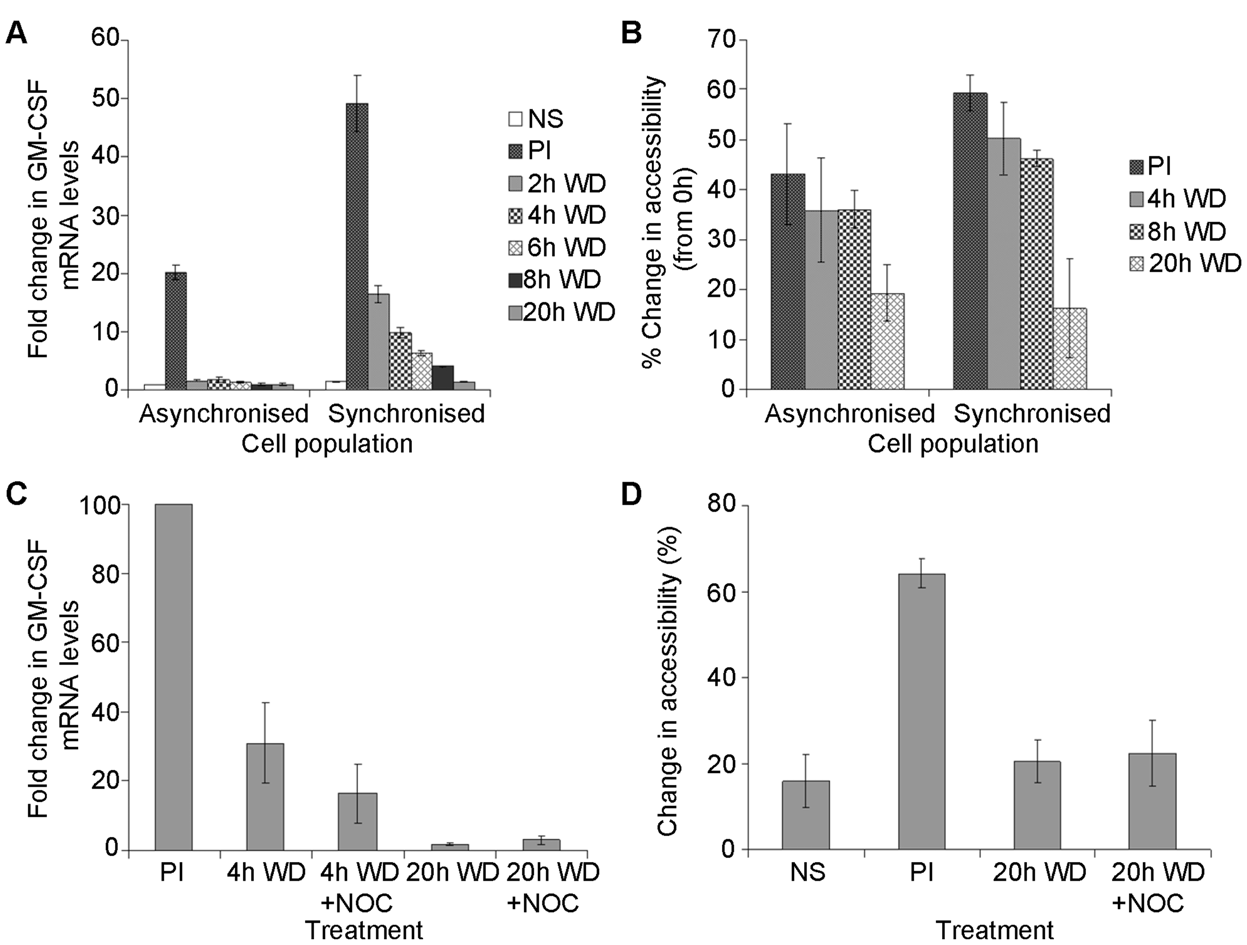
GM-CSF transcriptional down-regulation and promoter chromatin resetting is independent of the cell cycle. (A–B) Asynchronised and synchronised EL-4 T cells were left unstimulated (NS) or stimulated with PI for 4 h (PI) then the stimulus withdrawn (WD) for the indicated times. GM-CSF mRNA levels relative to GAPDH were then determined by RT-qPCR (A) and promoter accessibility to MNase was determined by CHART-PCR (B). The mean and standard error of three independent experiments is shown. (C) GM-CSF mRNA levels relative to GAPDH were determined in EL-4 T cells stimulated with PI for 4 h (PI) and then the stimulus withdrawn (WD) for 4 h or 20 h, with or without nocodazole (NOC), as indicated. GM-CSF mRNA levels are shown relative to the PI sample which was set at 100%. (D) GM-CSF promoter accessibility to MNase was determined in EL-4 T cells treated as indicated. The mean and standard error of five independent experiments is shown (C–D).

In the asynchronized cell population GM-CSF mRNA levels increased dramatically following PI stimulation for 4h, as observed previously (Figure 2A), then declined rapidly after withdrawal of the activating stimulus, reaching basal levels 4 h after stimulus withdrawal (Figure 2A). In the synchronized population much higher transcript levels were induced after 4h PI stimulation, compared to the asynchronized population (Figure 2A). This suggests the GM-CSF promoter may be more permissive to stimulation when in G1 stage of the cell cycle. While transcript levels declined substantially by 2 h after stimulus withdrawal, in the synchronized population they did not return to basal levels until 20 h post-stimulation (Figure 2A), suggesting this was independent of DNA replication which had occurred by 8 h.

Accessibility of the GM-CSF promoter was then examined using CHART-PCR to monitor histone deposition at the promoter in the asynchronized and synchronized cell populations. Following stimulus withdrawal, promoter accessibility declined progressively in both populations, but remained elevated at 20 h (Figure 2B), despite cell division having occurred by this time. This suggests that DNA replication and cell division were not involved in resetting the promoter chromatin. However, higher levels of accessibility were seen in the synchronized population (Figure 2B), which along with the higher transcript levels in this cell population, suggest that the cell cycle may influence gene activation.

To confirm that cell division was not necessary for promoter chromatin resetting, cells were stimulated with PI for 4 h then treated with an inhibitor of the cell cycle, nocodazole, to prevent cell division. Nocodazole depolymerizes microtubules and thus prevents cell division from completing. Cells were stimulated with PI for 4 h then either left untreated or treated with nocodazole following stimulus withdrawal. The ability of nocodazole to block the cell cycle resulting in accumulation of cells in G2/M phase was confirmed using flow cytometry (data not shown). Blocking cell division had no impact upon chromatin resetting with the decline in both GM-CSF mRNA levels (Figure 2C) and promoter accessibility (Figure 2D) upon stimulus withdrawal occurring similarly in both untreated and nocodazole treated cells.

Put together these data suggest that transcriptional down-regulation of the GM-CSF gene upon stimulus withdrawal, and the associated reassembly of histones at the gene promoter occur independently of DNA replication and cell division.

#### Depletion of NF-κB proteins and RNA polymerase II occurs concomitantly with histone reassembly at the GM-CSF and IL-2 promoters

**Figure 3:**
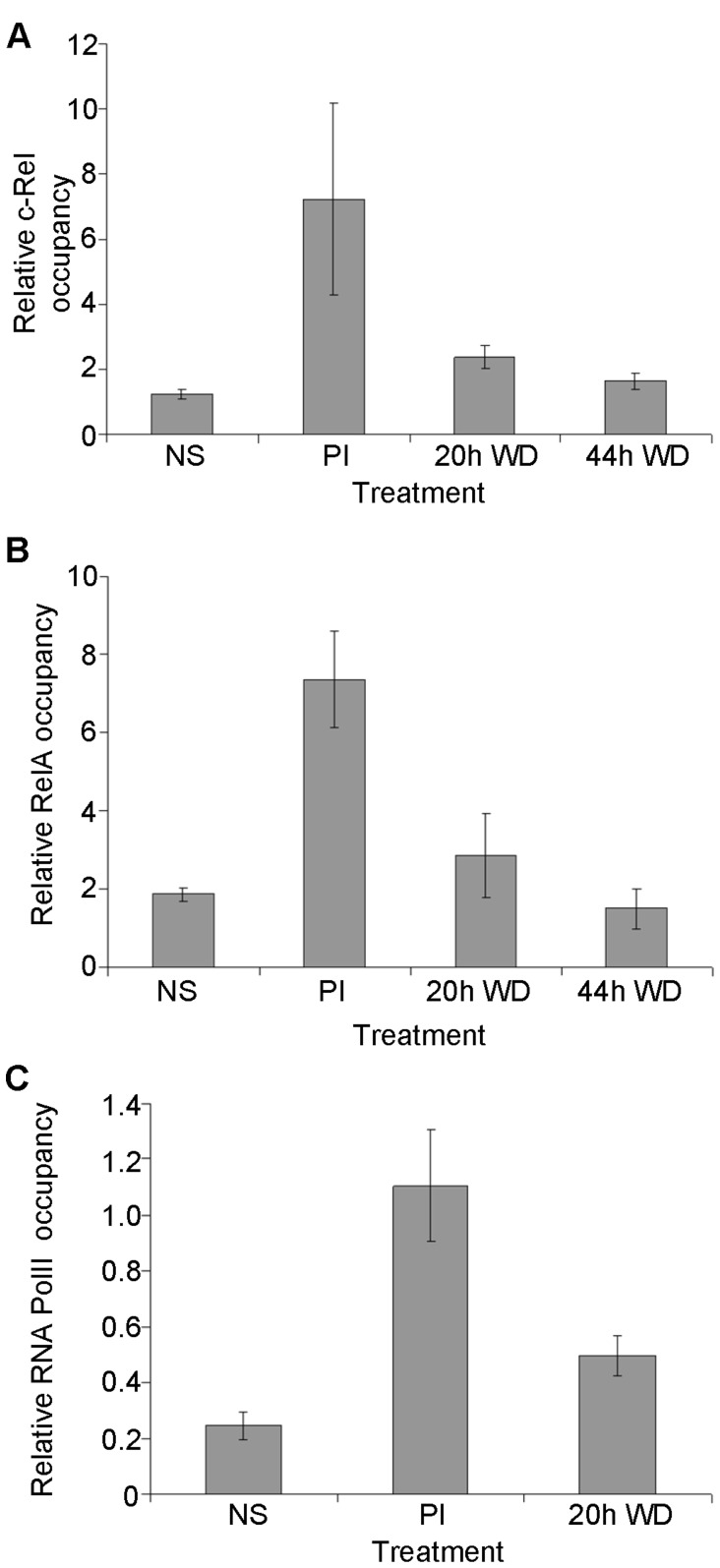
NF-κB and RNA Polymerase II occupy the active GM-CSF promoter. (A) c-Rel (B) RelA and (C) RNA polymerase II occupancy was determined at the GM-CSF promoter by ChIP analysis of unstimulated EL-4 T cells (NS), cells stimulated for 4 h with PI (PI) and cells in which the stimulus was withdrawn (WD) for the indicated times. Occupancy levels are shown relative to the inactive rhodopsin promoter. The mean and standard error of three independent experiments is shown.

We have previously demonstrated that the NF-κB transcription factor family, and particularly c-Rel, is essential for both chromatin remodeling events at the promoter and activation of the GM-CSF gene in EL-4 T cells upon T cell activation [11]. To determine whether NF-κB proteins may also influence chromatin resetting following stimulus removal, ChIP analysis was used to monitor c-Rel and RelA binding to the GM-CSF promoter. Little c-Rel or RelA were detected at the promoter in unstimulated EL-4 T cells, but both c-Rel and RelA became associated with the GM-CSF promoter following 4 h stimulation with PI, as expected (Figure 3A and 3B). Following withdrawal of the activating stimulus, c-Rel (Figure 3A) and RelA (Figure 3B) occupancy at the promoter diminished, with low levels detected at 20 h and a return to basal levels observed at 44 h. Therefore occupancy of NF-κB proteins at the promoter mirrors promoter accessibility following stimulus withdrawal (Figure 1D). This suggests that histone redeposition at the promoter may be dependent on the removal of NF-κB transcription factors.

We then examined whether depletion of NF-κB transcription factors from the promoter coincided with loss of the transcription machinery. RNA polymerase II is recruited to the GM-CSF promoter upon stimulation with PI for 4 h (Figure 3C). Upon withdrawal of the activating stimulus, RNA polymerase II occupancy declines, but remains elevated above basal levels at 20 h post stimulus withdrawal (Fig 3C). The depletion of RNA polymerase II from the GM-CSF promoter coincides with the kinetics of histone H3 redeposition at the GM-CSF promoter following transcriptional down-regulation (Figure 1E) and with the depletion of NF-κB transcription factors.

**Figure 4:**
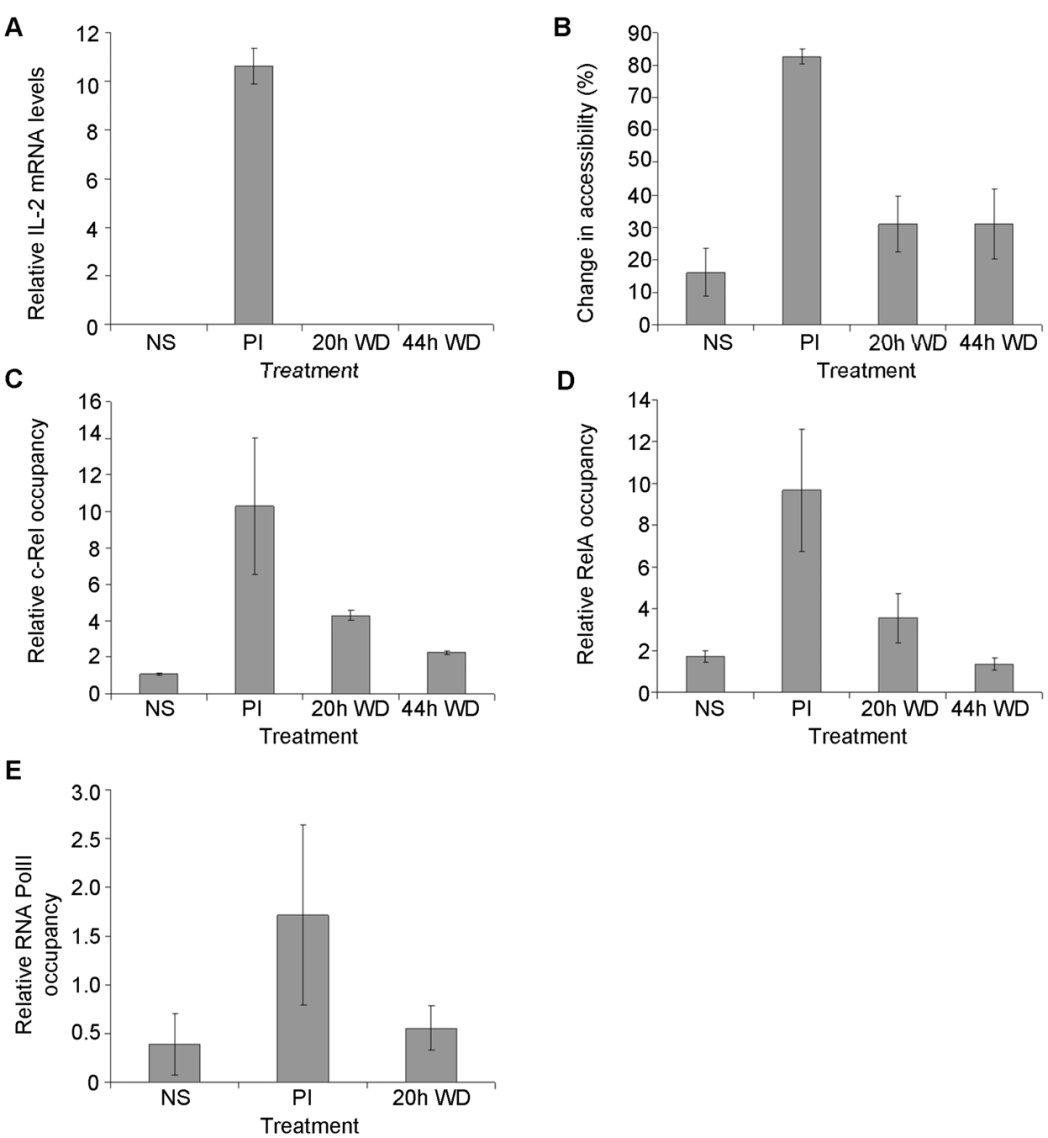
NF-κB and RNA Polymerase II are associated with the active IL-2 promoter. (A) IL-2 mRNA levels relative to GAPDH were determined in EL-4 T cells either left untreated or treated with PI for 4 h (PI), then the stimulus withdrawn (WD) for the indicated times. (B) IL-2 promoter accessibility was determined by CHART-PCR in cells treated as in (A). (C–E) c-Rel (C), RelA (D) and RNA polymerase II (E) occupancy was determined at the IL-2 promoter by ChIP in cells treated as indicated. Occupancy levels are shown relative to the inactive rhodopsin promoter. The mean and standard error of three independent experiments (A–D) or mean and standard deviation of two experiments (E) is shown.

To determine whether the mechanism of chromatin resetting observed at the GM-CSF promoter also operates at other inducible gene promoters in T cells, we examined the IL-2 promoter, which also undergoes chromatin remodeling in response to T cell activation in a c-Rel dependent manner [18], [23], [24]. IL-2 mRNA levels increased dramatically following 4h PI stimulation, but had returned to basal levels within 20 h of stimulus withdrawal (Figure 4A). Accessibility of the promoter also increased dramatically following 4h PI stimulation, as seen previously [18], [23], and declined by 20 h stimulus withdrawal (Figure 4B). As with the GM-CSF promoter, occupancy of c-Rel (Figure 4C) and RelA (Figure 4D) at the IL-2 promoter mirrored chromatin accessibility, with levels of these proteins at the IL-2 promoter increasing following 4 h PI stimulation and then declining following stimulus withdrawal for 20 h or 44 h. RNA polymerase II was also recruited to the promoter following stimulation but had been depleted from the promoter within 20 h of stimulus removal, returning to basal levels by this time (Figure 4E).

Together, these data suggest that histone reassembly at the GM-CSF and IL-2 promoters occurs concomitantly with depletion of RNA polymerase II and NF-κB transcription factors.

### Nuclear Translocation of IκBα is Required for Depletion of c-Rel from the Nucleus and Transcriptional Down-regulation of the GM-CSF Promoter

**Figure 5:**
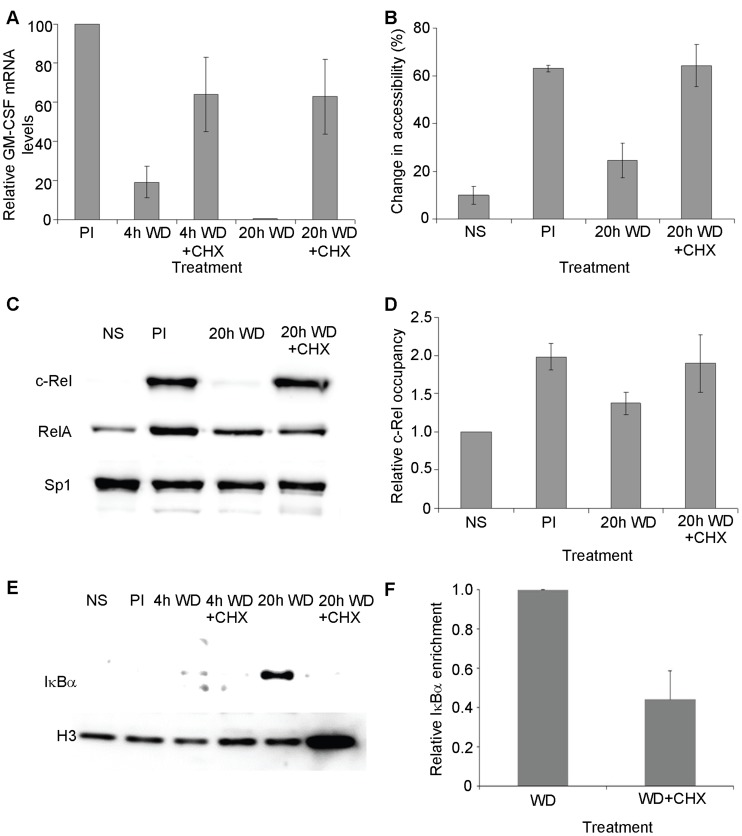
GM-CSF transcriptional down-regulation and promoter resetting is associated with nuclear depletion of c-Rel and accumulation of IκBα. (A–B) GM-CSF mRNA levels, relative to GAPDH (A) and promoter accessibility to MNase (B) was determined in unstimulated EL-4 T cells (NS), cells stimulated with PI for 4 h (PI) and cells in which the stimulus was withdrawn (WD) for the indicated times, or withdrawn for the indicated times in the presence of cycloheximide (CHX), as indicated. mRNA levels are shown in (A) relative to PI treated sample which was set at 100%. The mean and standard error of three independent experiments is shown. (C) Nuclear extracts of EL-4 T cells treated as indicated were subjected to western analysis with the indicated antibodies. (D) c-Rel occupancy at the GM-CSF promoter was determined by ChIP analysis in EL-4 T cells treated as indicated. c-Rel occupancy was normalised to levels in unstimulated cells (NS). The mean and standard error of four independent experiments is shown. (E) Nuclear extracts from EL-4 T cell treated as indicated were subjected to western analysis with the indicated antibodies. (F) IκBα occupancy at the GM-CSF promoter was determined by ChIP analysis of EL-4 T cells stimulated with PI for 4 h and then the stimulus withdrawn for 20 h in the absence or presence of CHX, as indicated. IκBα occupancy was normalized to levels in cells in which stimulus was withdrawn in the absence of CHX. The mean and standard error of three independent experiments is shown.

To further elucidate the process of chromatin resetting at the GM-CSF promoter following stimulus withdrawal, the requirement for new protein synthesis in nucleosome reassembly was explored. We have previously shown that new protein synthesis is essential for the chromatin remodeling events that precede GM-CSF and IL-2 gene activation 5,13. Therefore the effect of the protein synthesis inhibitor cycloheximide on GM-CSF mRNA levels and histone deposition following stimulus withdrawal was examined. Cells were stimulated for 4h with PI, then the stimulus withdrawn either in the absence or presence of cycloheximide. Cycloheximide treatment inhibited both the decline in GM-CSF mRNA levels (Figure 5A) and the decrease in promoter accessibility (Figure 5B) normally observed following stimulus withdrawal. These data suggest that protein synthesis is essential for transcriptional down-regulation and histone deposition at the GM-CSF promoter following stimulus withdrawal.

Next the effect of cycloheximide treatment on NF-κB proteins c-Rel and RelA was examined by western blotting. Little c-Rel or RelA was detected in the nucleus prior to stimulation, and as expected upon treatment with PI for 4 h, c-Rel and RelA accumulated in the nucleus (Figure 5C). Following stimulus withdrawal, NF-κB proteins were depleted from the nucleus, with no c-Rel and reduced RelA detected in the nucleus 20 h after stimulus withdrawal. While RelA depletion was still observed in cells treated with cycloheximide, no depletion of c-Rel from the nucleus was observed in cells treated with cycloheximide prior to stimulus removal (Figure 5C). Reprobing of the western blot for Sp1 which is constitutively present in the cell nucleus confirmed equal loading of proteins in each lane. Since the prolonged presence of c-Rel in the nucleus following cycloheximide treatment mirrors the failure of the promoter chromatin to reassemble histones following stimulus withdrawal, ChIP analysis was then used to examine c-Rel occupancy at the promoter following stimulus withdrawal in the presence of cycloheximide. This analysis showed that as before, c-Rel became associated with the promoter following stimulation, and while there was a decline in c-Rel occupancy at the promoter following stimulus withdrawal, this decline in c-Rel occupancy was not observed when the stimulus was removed in the presence of cycloheximide (Figure 5D).

To investigate the mechanism by which cycloheximide treatment results in retention of c-Rel in the nucleus, the effect of cycloheximide treatment on IκB proteins was examined. While regulation of NF-κB through interactions with IκB proteins in the cytoplasm has been well described [1], shuttling of IκBα between the cytoplasm and nucleus has also been demonstrated, with the nuclear translocation of IκBα reported to influence NF-κB binding [27], [28]. IκBα expression in the cell nucleus was therefore examined upon stimulus withdrawal. No IκBα was detected in the nucleus of unstimulated EL-4 T cells or cells stimulated for 4 h with P/I, but IκBα accumulated in the nucleus following stimulus withdrawal, with low levels detected in the nucleus 4 h post-stimulus withdrawal and increasing by 20 h, as determined by western blotting (Figure 5E). Therefore the accumulation of IκBα in the cell nucleus following stimulus withdrawal correlates with depletion of c-Rel from the GM-CSF gene promoter (Figure 5D) and resetting of the chromatin (Figure 5B). Further, treatment of cells with cycloheximide during stimulus withdrawal, which resulted in retention of c-Rel in the nucleus (Figure 5C) prevented accumulation of IκBα in the nucleus (Figure 5E). Reprobing of the western blot for H3 confirmed equal loading of proteins, but further demonstrated that H3 nuclear levels actually increase upon cycloheximide treatment, suggesting that lack of histones is not preventing resetting of the chromatin in the presence of cycloheximide. IκBα has been reported to target NF-κB proteins bound to gene promoters [29] and therefore enrichment of IκBα at the GM-CSF promoter was examined by ChIP, with increased IκBα detected at the promoter following stimulus withdrawal compared to cells in which the stimulus was withdrawn in the presence of cycloheximide (Figure 5F).

Together this data suggests that upon stimulus withdrawal IκBα accumulates in the cell nucleus, binding to c-Rel at target genes, leading to depletion of c-Rel from the nucleus and subsequent transcriptional down-regulation of c-Rel target genes.

#### Nuclear degradation of c-Rel results in termination of GM-CSF gene activation in response to stimulus withdrawal

**Figure 6:**
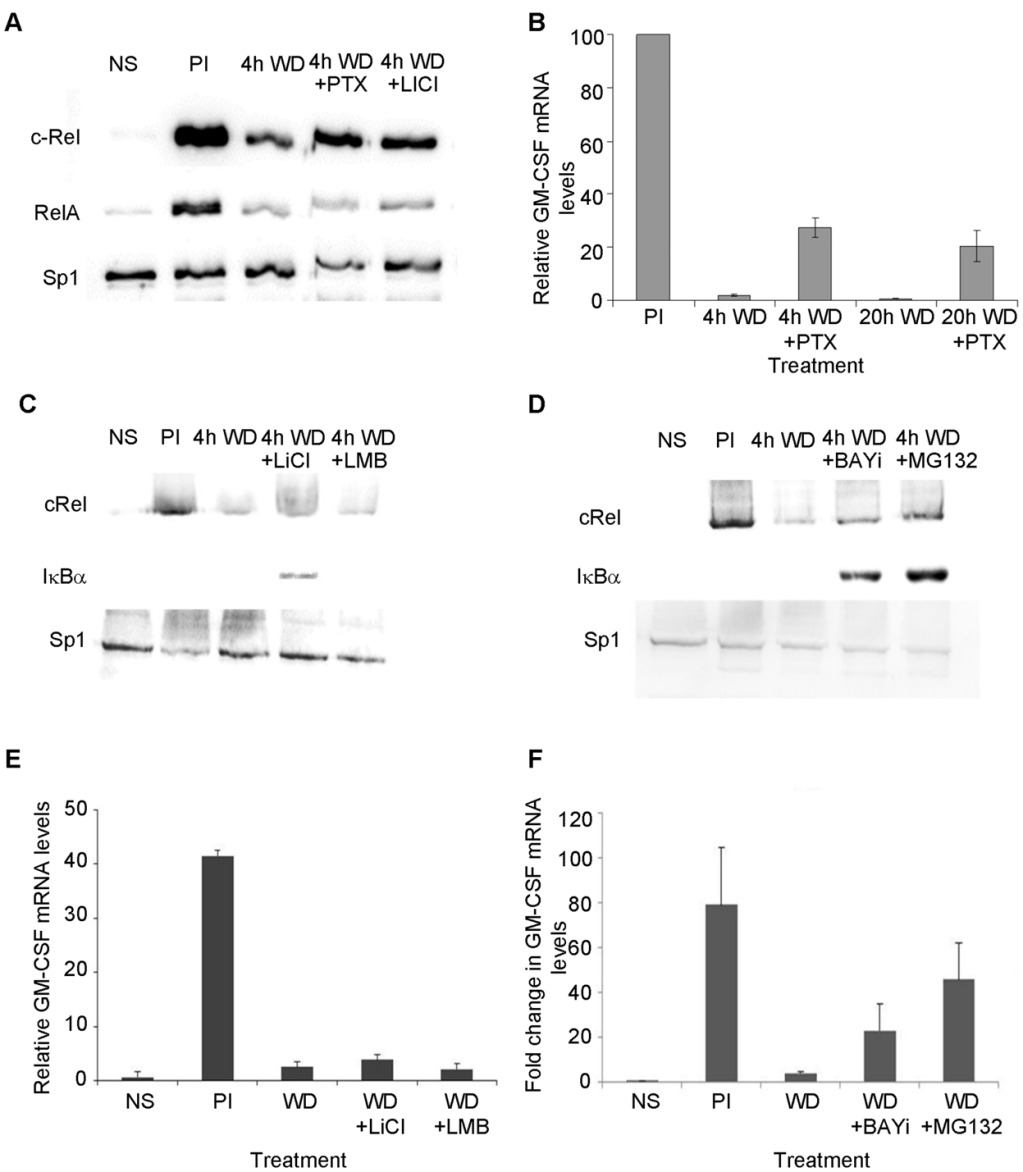
Transcriptional down-regulation of the GM-CSF gene is dependent on the nuclear depletion of c-Rel. (A) Nuclear extracts from EL-4 T cells either left unstimulated (NS), stimulated with PI for 4 h (PI), the stimulus withdrawn for 4 h (WD) or the stimulus withdrawn in the presence of pentoxifylline (PTX) or lithium chloride (LiCl), were subjected to western analysis with the indicated antibodies. (B) GM-CSF mRNA levels relative to GAPDH were determined in cells treated as indicated. mRNA levels are shown relative to the 4 h PI sample which was set at 100%. The mean and standard error of three independent experiments is shown. (C–D) Nuclear extracts from EL-4 T cells either left unstimulated (NS), stimulated with PI for 4 h (PI), the stimulus withdrawn (WD) for 4 h, or the stimulus withdrawn in the presence of lithium chloride (LiCl) or leptomycin B (LMB) (C) or BAY 11-7082 (BAYi) or MG132 (D) were subjected to western analysis with the indicated antibodies. (E–F) GM-CSF mRNA levels relative to GAPDH were determined in cells treated as indicated. The mean and standard deviation of two independent experiments is shown.

We have previously demonstrated using c-Rel^−/−^ mice that c-Rel is required for chromatin remodeling at the GM-CSF promoter [11], however because of its requirement for chromatin remodeling and gene activation, these mice are not useful to examine termination of the gene response. Therefore following stimulation, cells were treated with pentoxifylline, which has previously been shown to prevent nuclear accumulation of c-Rel, but not RelA in response to stimulation [30] and prevent chromatin remodeling at the GM-CSF promoter [11]. Cells were also analyzed in the presence of lithium chloride, which has been shown previously to prevent NF-κB-dependent gene activation [31]. Cells were either, left unstimulated, stimulated for 4 h with PI, then the stimulus withdrawn or the stimulus withdrawn in the presence of pentoxifylline or lithium chloride. As previously observed, upon PI stimulation c-Rel and RelA accumulated in the nucleus, and levels decreased upon stimulus withdrawal for 4 h (Figure 6A). While stimulus withdrawal in the presence of pentoxifylline or lithium chloride did not prevent depletion of RelA from the nucleus, depletion of c-Rel was partially inhibited (Figure 6A). GM-CSF mRNA levels were then monitored in cells treated with pentoxifylline during stimulus withdrawal. As before a decrease in GM-CSF mRNA levels was observed following stimulus withdrawal, however this was inhibited by treatment of cells with pentoxifylline (Figure 6B). Pentoxifylline had a similar effect on IL-2 mRNA levels (data not shown).

To further investigate the mechanism of c-Rel depletion from the nucleus following stimulus withdrawal, IκBα levels were examined in the nucleus in lithium chloride treated cells. While only low levels of IκBα could be detected in the nucleus following stimulus withdrawal for 4 h, increased IκBα was detected in the nucleus at this time in the presence of lithium chloride (Figure 6C). GM-CSF mRNA levels were then monitored in these cells, with lithium chloride treatment found to have no effect on transcriptional down-regulation of the GM-CSF gene following stimulus withdrawal (Figure 6E).

Put together these data suggest that nuclear accumulation of IκBα is required for the inactivation of c-Rel in the nucleus upon stimulus withdrawal. Two mechanisms have previously been proposed for IκBα-dependent depletion of NF-κB proteins from the nucleus; export of the proteins from the nucleus or degradation of the proteins within the nucleus via the proteasome. To investigate these possibilities, cells were treated with leptomycin B, which inhibits Crm1-dependent nuclear export of IκBα [32]. Leptomycin B treatment did not prevent nuclear depletion of c-Rel following stimulus withdrawal or affect nuclear accumulation of IκBα (Figure 6C). Further leptomycin B did not affect transcriptional down-regulation of the GM-CSF gene in response to stimulus withdrawal (Figure 6E). In contrast treatment of cells with the proteasome inhibitors BAY11-7082 and MG132 resulted in increased accumulation of IκBα in the nucleus following stimulus withdrawal and partially inhibited the depletion of c-Rel from the nucleus in these cells (Figure 6D). In keeping with this both of these proteasome inhibitors partially prevented transcriptional down-regulation of GM-CSF following stimulus withdrawal (Figure 6F).

These data therefore suggest that upon stimulus withdrawal, IκBα accumulates in the cell nucleus removing c-Rel from the GM-CSF gene promoter, and targeting it for degradation via the proteasome within the nucleus.

## Discussion

Cytokine genes are generally maintained in a repressed state in resting T cells, with T cell activation resulting in a dramatic but transient increase in expression of a large number of cytokine genes. Activation of these genes requires complex interaction between transcription factors and the chromatin environment and while the chromatin changes that facilitate activation of inducible genes are now relatively well understood, comparatively less is known about how gene responses are terminated. We have shown here and previously [13] that the GM-CSF promoter is hyperacetylated in T cells. During activation of the GM-CSF and IL-2 genes in response to T cell stimulation, the hyperacetylated histones are depleted from the gene promoter to facilitate gene transcription [24]. The data presented here, together with our previous studies, demonstrate that following withdrawal of the activating stimulus, histones are reassembled at the promoter, as measured by the increasing occupancy of histone H3 at the promoter and a decline in DNA accessibility [24]. However, the mechanism underlying the transcriptional down-regulation of these genes has not been examined. One potential mechanism by which histones could be replaced onto the DNA would be during DNA replication. However, in this study we show that redeposition of histones at the GM-CSF promoter is independent of DNA replication or the cell cycle. Insight into the process of chromatin reassembly at inducible promoters has been largely limited to investigations in yeast [33], [34], [35], [36] and studies of the yeast PHO5 gene have similarly found that DNA replication is not required for nucleosome reassembly at that gene promoter during transcriptional down-regulation [34]. Furthermore, when nucleosome reassembly was prevented at the PHO5 promoter, the nucleosome depleted state could be inherited through DNA replication, and transcriptional activity was maintained despite the absence of transcription factors from the promoter [37]. These findings suggest that the mechanism of histone redeposition at gene promoters during transcriptional down-regulation is distinct from the mechanisms which operate to deposit histones on replicating DNA.

The major finding of this study however is that chromatin resetting and transcriptional down-regulation of the GM-CSF and IL-2 genes is dependent on the removal of the NF-κB transcription factor c-Rel from the gene promoters. Regulation of the GM-CSF and IL-2 genes in T cells by the NF-κB transcription factors RelA, p50 and c-Rel, has been well described [10], but we have previously found that chromatin remodelling events at the promoters that facilitate gene activation are specifically dependent on the c-Rel family member [5], [11]. Similarly we show here that while activation of the IL-2 and GM-CSF genes involves association of both c-Rel and RelA with the gene promoters and that histone redeposition at the promoters occurs concomitantly with the disassociation of these factors from the promoter, resetting of the promoter chromatin is specifically dependent on depletion of c-Rel from the nucleus. This data is consistent with a model in which c-Rel binding is in direct competition with histone deposition at a gene promoter.

Regulation of NF-κB activity is complex with the role of the inhibitory IκB proteins in NF-κB regulation well-described [1]. IκB is present in the cytoplasm of resting T cells in complex with NF-κB proteins. Upon T cell stimulation IκB is phosphorylated by IKK and subsequently ubiquitinated and degraded via the proteasome, allowing NF-κB translocation to the nucleus. The IκB genes themselves are targets of NF-κB resulting in upregulation of IκB, which upon removal of the activating stimulus results in accumulation of IκB in the cytoplasm once more. In addition, a nuclear role for IκBα in regulating NF-κB has more recently become apparent [38], with shuttling of IκBα between the cytoplasm and nucleus previously described in a range of cell types, including T cells [27], [28], [39]. Here we show that IκBα accumulates in the nucleus of T cells following the removal of the activating signal. Further, this is required for depletion of c-Rel from the nucleus and termination of gene responses to the activating signal. In addition we detected association of IκBα with the GM-CSF promoter suggesting that upon translocation to the nucleus IκBα becomes associated with NF-κB bound to target genes. In support of this notion, IκBα has previously been found to target DNA-bound RelA for degradation in fibroblasts [29]. Interestingly though, here nuclear accumulation of IκBα was required for removal of c-Rel but not RelA from the nucleus, with depletion of RelA from the nucleus upon stimulus withdrawal occurring regardless of the presence of IκBα. This highlights the complexity of NF-κB regulation; demonstrating differential regulation of NF-κB family members, but further, NF-κB regulation is increasingly being found to be cell-type as well as stimuli-specific. For example, while RelA depletion from the nucleus of T cells was found here to be independent of IκBα, nuclear association of IκBα with RelA has previously been shown to be important in regulating its DNA binding in macrophages and neutrophils [40], [41]. Furthermore, the CRM1 inhibitor leptomycin B was found here to have no effect on the nuclear accumulation of IκBα or depletion of c-Rel following stimulus withdrawal, whereas the CRM1 nuclear export pathway has been demonstrated to be important in regulating IκBα nuclear activity in other circumstances [32], [41]. This suggests that depletion of c-Rel following withdrawal of T cell activating signals occurs via a mechanism other than nuclear export in association with IκBα. An alternative mechanism, involving targeting of RelA for nuclear degradation by the proteasome has also been described [29]. Such a mechanism may be operating to control c-Rel activity in this instance and is supported by our data demonstrating accumulation of IκBα in the nucleus following treatment with proteasome inhibitors, resulting in delayed depletion of c-Rel and delayed transcriptional down-regulation of the GM-CSF promoter. In contrast, LiCl treatment, which also led to accumulation of IκBα in the nucleus and delayed depletion of c-Rel did not affect transcriptional down-regulation of the GM-CSF gene. However LiCl treatment is likely affecting the NF-κB pathway by an alternative mechanism, and for example has been found to inhibit RelA activity by blocking its phosphorylation by GSK-3β [31].

The data presented here demonstrated recruitment of RNA polymerase II to the GM-CSF and IL-2 gene promoters concomitant with c-Rel and RelA binding, and similarly removal of these proteins from the promoter was mirrored by a decrease in RNA polymerase II binding. While the focus here was on histone changes and transcription factor binding at the promoter, there is evidence, particularly from studies in yeast that histone methylation changes are intimately linked with RNA polymerase II activity [42]. The histone methyltransferases Set1 and Set2, that methylate H3K4 and H3K36, respectively, have been demonstrated to interact with RNA polymerase II and influence transcription. H3K4 trimethylation is associated with the 5′ region of actively transcribed genes, due to the association of Set1 with RNA polymerase II. However, at least in yeast components of the Set1 complex have also been linked with transcriptional termination [43]. In contrast Set2 associates with elongating RNA polymerase II and is enriched through actively or recently transcribed genes [44]. Examination of histone modifications, particularly histone methylation, across the GM-CSF and IL-2 genes during gene activation and following stimulus removal may therefore provide insight into the role of these histone modifying complexes and histone methylation in regulating RNA polymerase II activity and inducible gene expression.

In conclusion, the data presented here demonstrate that the nuclear accumulation of IκBα is critical in terminating responses of the cytokine genes GM-CSF and IL-2 in activated T cells, following the withdrawal of the activating stimulus. Activation of both of these genes has been found to be dependent on depletion of histones from the gene promoters, which is critically regulated by c-Rel. The data presented here is consistent with a mechanism in which termination of the gene response is dependent on inactivation of c-Rel in an IκBα dependent manner, involving targeting of the proteins for degradation within the nucleus.
